# A lovastatin-elicited genetic program inhibits M2 macrophage polarization and enhances T cell infiltration into spontaneous mouse mammary tumors

**DOI:** 10.18632/oncotarget.1376

**Published:** 2013-10-26

**Authors:** Emilia Mira, Lorena Carmona-Rodríguez, Manuel Tardáguila, Iñigo Azcoitia, Alicia González-Martín, Luis Almonacid, Josefina Casas, Gemma Fabriás, Santos Mañes

**Affiliations:** ^1^ Department of Immunology and Oncology, Centro Nacional de Biotecnología/CSIC, Madrid, Spain,; ^2^ Department of Cell Biology, School of Biology, Universidad Complutense de Madrid, Madrid, Spain,; ^3^ Department of Biomedicinal Chemistry, Catalan Institute of Advanced Chemistry/CSIC, Barcelona, Spain; ^4^ Present address: The Scripps Research Institute, Department of Immunology and Microbial Science, La Jolla, CA

**Keywords:** inflammation, tumor immunity, macrophage polarization, angiogenesis, vascular normalization, myeloid infiltration, lymphoid infiltration, statins

## Abstract

Beyond their ability to inhibit cholesterol biosynthesis, the statins have pleiotropic effects that include anti-inflammatory and immunomodulatory activities. Statins could have clinical utility, alone or in combination with other chemotherapeutics, in the treatment of cancer. The mechanisms that underlie the anti-tumor activity of the statins are nonetheless poorly defined. No studies have analyzed how they alter the tumor-associated leukocyte infiltrate, a central factor that influences tumor stroma and cancer evolution. Here we used *HER2/neu* transgenic (Tg-neu) mice to analyze the effect of lovastatin (Lov) on the inflammatory reaction of spontaneous mammary tumors. Lov treatment of tumor-bearing Tg-neu mice did not alter growth of established tumors, but significantly reduced the number of new oncogenic lesions in these mice. Moreover, Lov inhibited the growth of newly implanted Tg-neu tumors in immunocompetent but not in immunodeficient mice. We found that Lov enhanced tumor infiltration by effector T cells, and reduced the number of immunosuppressive and pro-angiogenic M2-like tumor-associated macrophages (TAM). Concomitantly, the drug improved the structure and function of the tumor vasculature, measured as enhanced tumor oxygenation and penetration of cytotoxic drugs. Microarray analysis identified a Lov-elicited genetic program in Tg-neu tumors that might explain these effects; we observed Lov-induced downregulation of placental growth factor, which triggers aberrant angiogenesis and M2-like TAM polarization. Our results identify a role for lovastatin in the shaping and re-education of the inflammatory infiltrate in tumors, with functional consequences in angiogenesis and antitumor immunity.

## INTRODUCTION

The progression or inhibition of a tumor is intimately linked to the integration of complex signals delivered from its microenvironment. Evidence indicates that immune cells in the tumor vicinity are major regulators of the outcome. Tumor infiltration by cells of the adaptive immune arm is associated with good prognosis in glioblastoma, colon and ovarian cancers [1-3]; in contrast, massive accumulation of cells of the innate immune system, particularly macrophages, is linked to poor prognosis [4-7]. Tumor-associated macrophages (TAM) nonetheless have contrasting activities, depending on their differentiation state. Progressing tumors skew TAM differentiation towards an alternatively activated (M2-like) state that induces angiogenesis and aids tumor cell evasion of antitumor immunity. In non-progressing or regressing tumors, however, TAM tend to a classic, pro-inflammatory (M1-like) program that promotes adaptive immune responses and tumor lysis [5, 6]. The ability to regulate the type of inflammatory infiltrate as well as its differentiation program is thus central to the way that inflammation affects tumor evolution.

Given the strong influence of inflammation on tumor biology, anti-inflammatory drugs are being screened for antitumor activity. The statins are a family of inhibitors of the 3-hydroxy-3-methylglutaryl coenzyme A (HMG-CoA) reductase enzyme, which converts acetyl-CoA into mevalonic acid. Since HMG-CoA reductase catalyzes the rate-limiting step in the mevalonate pathway of cholesterol biosynthesis in the liver, it was thought that the major clinical benefit of statins was to reduce cholesterol levels in the bloodstream [8]; statins are thus in wide clinical use for the prevention and treatment of cardiovascular disease [9]. Nonetheless, mevalonate is also the precursor of isoprenoid compounds, which are substrates for post-translational modification of many proteins involved in cell signaling. Blockade of isoprenoid synthesis might explain the pleiotropic effects reported for statins in extrahepatic tissues, including inhibition of pathogen infection as well as anti-inflammatory and immunomodulatory activities [10-15].

Many studies indicate that statins might also have anti-tumorigenic activity, including inhibition of angiogenesis [16, 17] and direct cytotoxicity of tumor cells [18-20]. Breast cancer cells bearing mutated p53 upregulate the mevalonate pathway to disrupt mammary acinar morphology and promote tumorigenesis, suggesting that statins can be useful for tumors with mutations in this suppressor gene [21]. Although these studies and some epidemiological analyses suggest a preventive role for statins in human cancer, randomized clinical trials indicate that statins are not potent anti-cancer agents in monotherapy-based regimes (reviewed in [22]). Statins can also potentiate other cytotoxic drugs [23-25], however, which prompted a number of ongoing phase I/II clinical trials to test whether statins improve chemotherapeutic effectiveness of other anti-cancer drugs in skin (NCT00966472), gastric (NCT01099085), prostate (NCT01220973), and breast cancers (NCT00354640) (http://www.clinicaltrials.gov). The mechanisms that underlie statin synergy with cytotoxic drugs are diverse and not fully known. Statins overcome tumor cell resistance to cytotoxic drugs by targeting multidrug resistance-associated proteins, and alleviate the secondary effects of chemotherapy in kidney and heart (reviewed in [25]). Recent evidence suggests that statins increase penetration of cytotoxic compounds into the tumor by regulating endothelial nitric oxide (NO) synthesis and oxidative stress; this in turn normalizes tumor blood vessel morphology, maturation and function [26]. Statins nonetheless induce pericyte apoptosis *in vitro* [27]; this apparent contradiction implies complexity in the way statins alter the tumor vasculature.

Although a major pleiotropic activity of statins is the regulation of immune and inflammatory responses, the relevance of these statin-mediated effects in cancer has not been studied in detail. Pravastatin was reported to downregulate expression of pro-inflammatory and pro-angiogenic factors, which correlated with tumor growth inhibition in syngeneic mice [17]. In experimental models of autoimmunity and chronic inflammation, statins provoke a shift in T cell polarization towards a Th2 phenotype, and increase regulatory T (Treg) cell differentiation and recruitment (reviewed in [14]. These activities could be thought to have a negative impact on the potential immune response to tumors, and thus promote oncogenesis and tumor progression. Whether statin treatment impairs immune function in tumor models has not been reported.

We administered the natural statin lovastatin (Lov) to transgenic FVB/N-Tg(MMTVneu) mice (Tg-neu), which overexpress the HER2/*neu* proto-oncogene and develop spontaneous mammary tumors. Compared to tumor graft models, in which implantation causes tissue damage and hence inflammation, Tg-neu tumors generate an inflammatory response that better resembles that of sporadic human tumors. Tg-neu mice develop an immune response to neu antigen, which is functionally suppressed as in human tumors [28]; the residual neu-specific T cell repertoire can be reactivated to restrict tumor growth [29]. We found that Lov treatment of tumor-bearing Tg-neu mice did not alter growth of established tumors, but significantly reduced the onset of new oncogenic lesions. Lov inhibited TAM polarization toward a pro-tumorigenic M2-like phenotype and increased T cell infiltration into the tumor. These changes paralleled the stabilization of tumor blood vessel structure; indeed, Lov treatment reduced tumor hypoxia and enhanced doxorubicin penetration into Tg-neu tumors. Expression profiling identified a genetic program elicited by Lov treatment in these tumors, which included downregulation of placental growth factor (PlGF), an inducer of vasculature abnormalization as well as M2-like TAM polarization in tumors [30]. These combined Lov activities might create a hostile inflammatory environment for the tumor, in which antitumor immunity dominates over immune evasion, explaining the reduced tumor multiplicity in Lov-treated Tg-neu mice.

## RESULTS

### Lovastatin treatment does not alter growth of established tumors but reduces appearance of new lesions

**Figure 1:**
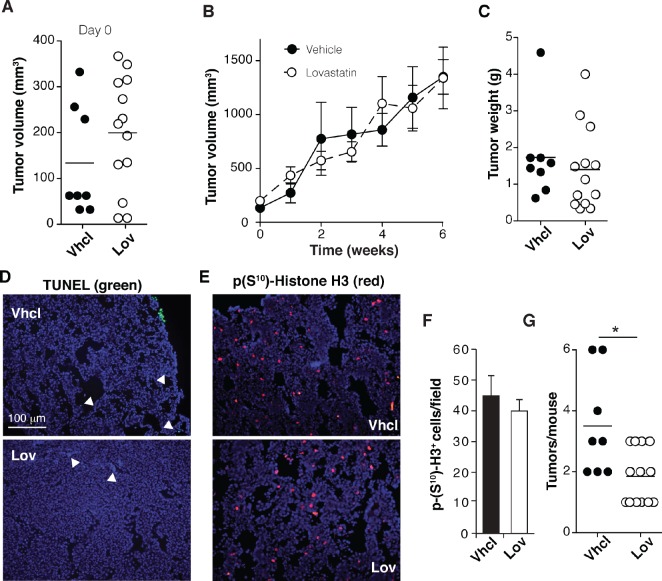
Lovastatin reduces tumor multiplicity in Tg-neu mice Tg-neu mice were assigned for Vhcl or Lov treatment after lump detection (*n* = 8, Vhcl; *n* = 11, Lov). (A) Volume of individual tumors at treatment onset. (B) Growth kinetics of primary Tg-neu tumors in Vhcl- or Lov-treated mice. (C) Weight of each initial tumor after sacrifice. (D, E) Representative images of sections from the initial tumor, stained for TUNEL (D) and p-H3^+^ (E); bar = 100 µm. (F) Quantification of images in *E* (*n* = 15 sections from 5 tumors/group; p = 0.35, Mann-Whitney test). (G) Tumor number for each mouse. *p <0.05, two-tailed Student's *t*-test.

Transgenic FVB/N-Tg(MMTVneu) mice (Tg-neu), which overexpress the *neu* protoncogene in the mammary gland and develop spontaneous mammary tumors, were randomly assigned for treatment with vehicle (Vhcl) or Lov as soon as lumps were detected by palpation (Fig. 1A). The Lov dose used (10 mg/Kg every 3 days, i.p.) is comparable to that for humans treated with 40 mg/day [31].

In this model, Lov injection did not affect growth kinetics of primary tumors (Fig. 1B) or their weight at endpoint (Fig. 1C) compared to Vhcl treatment. Immunohistochemical analysis showed no differences in the apoptotic cell fraction between Vhcl- and Lov-treated tumors (Fig. 1D; TUNEL^+^ cells/field, 0.37 ± 0.01 Vhcl vs. 0.29 ± 0.01 Lov, p = 0.7; *n* = 6/group). Although we generally noted a slight reduction in the proliferating cell fraction (phosphohistone H3; p-H3^+^) in tumors from Lov-treated mice, these differences were not significant (Fig. 1E, F; p = 0.5, *n* = 6/group). Macroscopic lung metastases were not detected in these mice.

Tg-neu-treated mice initially developed focal adenocarcinomas; multifocal lesions nonetheless appeared at longer latency periods. Although Lov treatment did not impair growth of the primary tumor, it significantly reduced tumor multiplicity (mean number of affected glands/mouse) compared to controls (Fig. 1G). At 6 weeks after initial lump detection, 100% of Vhcl-treated mice showed tumors in at least two mammary glands and 25% developed tumors in up to six; in contrast, 46% of Lov-treated mice had only one affected mammary gland, with a maximum of lesions in three glands (30% of the mice). Treatment of sporadic mammary tumors with high/medium Lov doses thus did not reduce tumor growth, but precluded promotion of new oncogenic lesions in the mammary gland.

### Lovastatin potentiates tumor chemotherapy by increasing drug delivery into tumors

**Figure 2:**
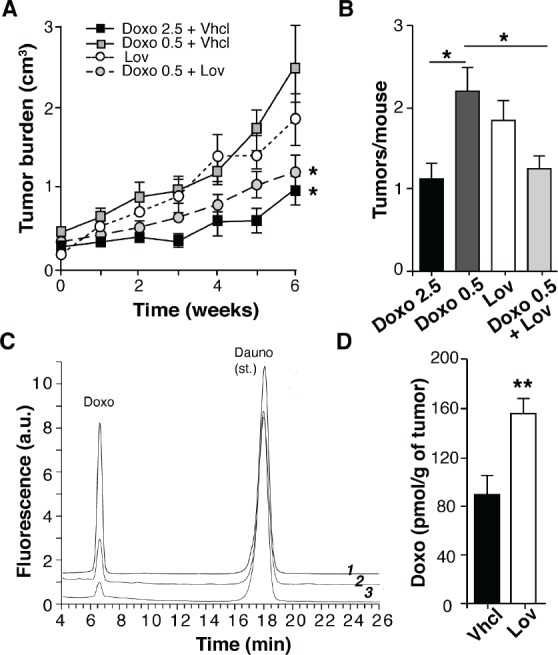
Lovastatin improves doxorubicin chemotherapy in Tg-neu mice (A) Growth kinetics of spontaneous Tg-neu tumors treated with Lov, Doxo+Vhcl or Doxo+Lov (*n* = 10/group; *, significant differences with the Doxo 0.5+Vhcl-treated group (p <0.05, repeated measures ANOVA with Dunnett post-test). (B) Mean tumor number in mice from *A* (p <0.05, one-way ANOVA with Dunnet post-test). (C) HPLC-FD profiles of a tissue extract containing Doxo and daunorubicin standards (*1*), and representative tumor samples from mice treated with 0.5 mg/Kg Doxo+Lov (*2*) or Doxo+Vhcl (*3*). (D) Doxo quantification in samples from *C* (*n* = 6; **p <0.01 Mann-Whitney test).

Statins can enhance the activity of several cytotoxic drugs, including the anthracyclins [25]. We thus tested whether Lov intensified the chemotherapeutic activity of doxorubicin (Doxo) in mice with spontaneous Tg-neu mammary tumors. Tg-neu tumors treated with a suboptimal Doxo dose (0.5 mg/Kg, twice weekly) or with Lov alone showed rapid growth despite treatment (Fig. 2A). Co-administration of Doxo (0.5 mg/Kg) + Lov significantly inhibited tumor growth at levels comparable to those found after 2.5 mg/Kg Doxo administration (Fig. 2A). In addition, Doxo+Lov treatment significantly reduced tumor multiplicity in Tg-neu mice (Fig. 2B).

The Lov-induced enhancement of Doxo chemotherapeutic activity might be due to potentiation of Doxo cytotoxic activity or to increased Doxo perfusion into the tumor parenchyma. To explore the latter hypothesis, we used high-performance liquid chromatography coupled with fluorescence detection (HPLC-FD) to analyze Doxo levels in extracts of tumors treated with Doxo (0.5 mg/Kg) +Vhcl or +Lov, using daunorubicin as an internal standard (Fig. 2C). Compared to Vhcl treatment, Lov co-administration significantly enhanced Doxo levels in tumors (Fig. 2D). Lov thus increased chemotherapy efficiency by improving delivery of this drug into the tumor.

### Lovastatin restores correct structure to tumor vasculature

**Figure 3:**
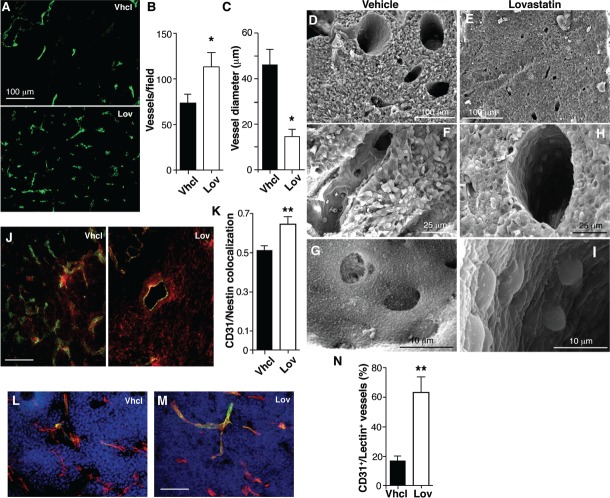
Lovastatin regulates Tg-neu tumor vascular phenotype (A) Anti-CD31 antibody staining of blood vessels in Vhcl- and Lov-treated tumors. (B,C) Quantification of vessel density and diameter from images as in *A* (*n* ≥10 slices from each of 4 tumors/condition). (D-I) Blood vessel ultrastructure in tumors from Vhcl- (*D, F, G*) and Lov-treated (*E, H, I*) mice. (J) Pericyte coverage of vessels (CD31^+^, green), detected by nestin staining (red) in Vhcl- and Lov-treated tumors; yellow, double-stained area. (K) Quantification of nestin and CD31 colocalization in *J*. (L,M) FITC-lectin-perfused Vhcl- (*L*) and Lov-treated (*M*) mice, co-stained for CD31. (N) Quantification of CD31^+^/lectin^+^ vessels in *L* and *M*. In all cases, *n* ≥30 from at least 3 tumors/condition. *B, C*, *K*, *N*: *p <0.05, **p<0.01 two-tailed Student's *t*-test.

Improved drug perfusion into tumors is associated with changes in tumor blood vessel structure and function; we thus tested whether Lov treatment affected angiogenesis in our model. Histochemical analysis showed clear differences in the number and morphology of CD31^+^ tumor blood vessels after Lov treatment (Fig. 3A). Vessels in Vhcl-treated tumors were less abundant (Fig. 3B) and their diameter was heterogeneous (Fig. 3C) compared to those in Lov-treated tumors, which were longer and thinner (Fig. 3C). Reduced vessel diameter in Lov-treated tumors compared to controls was also observed by scanning electron microscopy (SEM) (Fig. 3D, E). Ultrastructural analysis of Vhcl-treated tumors showed many of the phenotypic abnormalities of endothelial cells (EC) described in other tumor models, including irregular borders and discontinuities or gaps in the EC layer (Fig. 3F, G), which suggests EC hyperactivity. This contrasted with the regular, continuous, tightly packed EC in Lov-treated tumors (Fig. 3H, I), which lend an appearance of “smoothness” to the vessel lumen.

### Lovastatin increases pericyte coverage and tumor perfusion

Morphological irregularities in EC are often associated to poor coverage by mural cells [32]. Staining for CD31 and the intermediate filament nestin showed that Lov treatment significantly enhanced mural cell coverage (Fig. 3J, K). Analysis of tumor sections from mice perfused with FITC-lectin showed that Lov treatment also increased the percentage of vessels that double-stained for lectin and CD31 compared to controls (Fig. 3L-N). Lov treatment therefore improved tumor perfusion, probably by increasing vessel stability and maturation.

### Lovastatin improves tumor oxygenation

**Figure 4:**
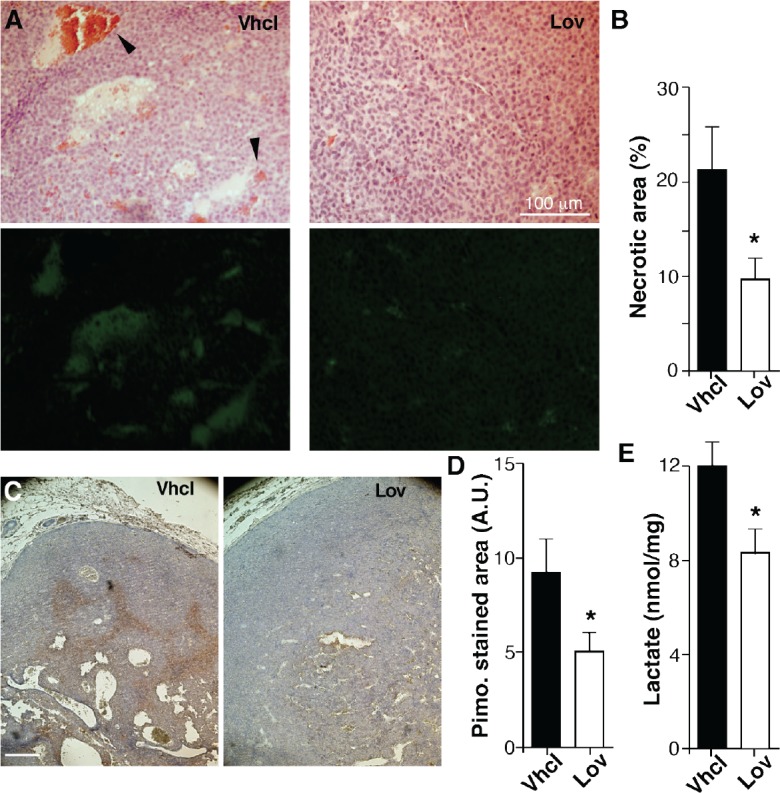
Lovastatin improves Tg-neu tumor oxygenation (A) Histological sections of necrotic areas in Vhcl- and Lov-treated tumors, hematoxylin/eosin-stained (top) or analyzed for autofluorescence (bottom). Arrowheads indicate “blood lakes”. (B) Quantification of necrotic areas in *A*. (C) Pimonidazole staining to detect hypoxic areas. (D) Quantification of pimonidizole-stained area in the sections in *C*. *B, D*: *(n* ≥5 sections from at least four distinct tumors/group; *p <0.05; Mann-Whitney test). (E) Lactate concentration in Vhcl- and Lov-treated tumors (*n* = 5 tumors/group; *p < 0.05, two-tailed Student's *t*-test).

Enhanced perfusion and increased blood vessel number should improve oxygenation of Lov-treated tumors. Hematoxylin/eosin staining showed that tumors from Vhcl-treated mice had large necrotic areas, which were not observed in those treated with Lov (Fig. 4A). Necrotic regions also showed enhanced autofluorescence (Fig. 4A); the extension of these fluorescent areas was significantly smaller in Lov-treated tumors (Fig. 4B). “Blood lakes”, which are associated with impaired vessel function and oxygenation [33], were usually found near the necrotic areas (Fig. 4A, arrowheads).

To further study tumor oxygenation, Vhcl- and Lov-treated mice received injections of the hypoxia marker pimonidazole (Fig. 4C). Pimonidazole-stained areas were larger (Fig. 4D) and more intensely labeled (not shown) in size-matched tumors from Vhcl- compared to Lov-treated mice. Consistent with enhanced tumor oxygenation, Lov treatment significantly reduced lactate levels (Fig. 4E), whose accumulation is linked to glycolytic metabolism in hypoxic tissues (Warburg effect). Lov treatment thus enhanced oxygenation of spontaneous mammary tumors in Tg-neu mice.

### Lovastatin downregulates PlGF expression in mammary tumors

To identify unique gene expression signatures responsible for Lov-induced vascular changes, we performed microarray analyses with mRNA isolated from tumors of Vhcl- and Lov-treated mice (*n* = 5/group). Genes showing a ±2-fold difference and a p-value <0.002 were considered to be differentially regulated by Lov. Based on these criteria, Lov treatment upregulated 39 (Table S1) and downregulated 49 genes (Table S2) compared to Vhcl; none of the differentially regulated genes were significant at a false discovery rate (FDR) <0.25. Based on their treatment-induced up- or downregulation or on biological significance, we selected 28 genes for assay by quantitative real-time (qRT)-PCR in a set of six independent tumors for each treatment condition (Table S1, S2); 75% of the upregulated and 95% of the downregulated genes were validated.

**Figure 5:**
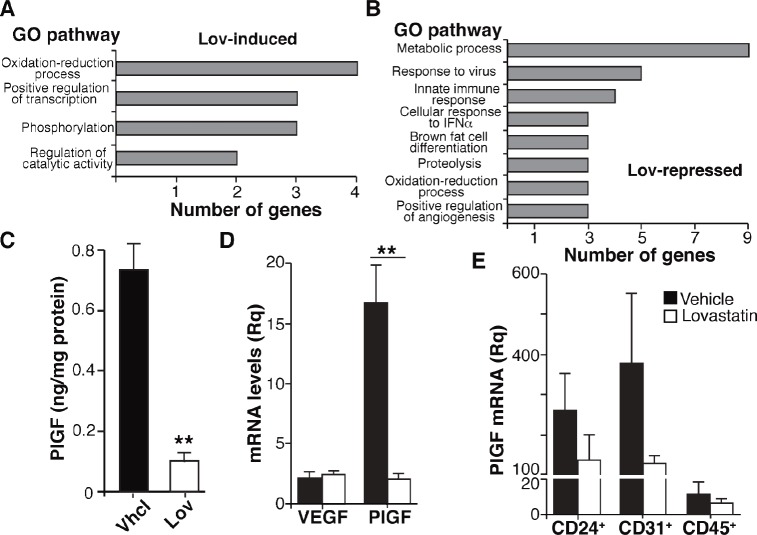
Lovastatin triggers a genetic program that targets angiogenesis, oxidative stress and inflammation (A,B) Main gene ontology processes significantly induced (*A*) or repressed (*B*) by Lov treatment (FDR <0.05). (C) PlGF levels in tumor extracts from Vhcl- or Lov-treated Tg-neu mice. Values show mean ± SEM of triplicates in one representative experiment of two (*n* = 6 tumors/group). (D) VEGF and PlGF mRNA levels in Vhcl- or Lov-treated Tg-neu tumors. Relative quantity was calculated using as reference the sample with the lowest VEGF or PlGF mRNA value. Mean ± SEM (*n* = 5). (E) PlGF mRNA quantification in CD24^+^, CD31^+^ and CD45^+^ cell populations isolated from Vhcl- or Lov-treated tumors (*n* = 4). *C*, *D*, ** p <0.01, two-tailed Student's t-test.

Gene ontology analysis of differentially regulated transcripts predicted a number of biological processes significantly altered by Lov treatment (Fig. 5A, B; FDR <0.05). Lov-affected processes included oxidation/reduction, which concurs with the changes in tumor oxygenation, metabolism, including the downregulation of the glycolytic enzyme 6-phosphofructokinase/fructose-2,6-bisphosphate (*pfkfb3*) gene, as well as the downmodulation of positive regulators of angiogenesis. We found that Lov downregulated the placental growth factor (*plgf*) gene, whose elevation is associated with the abnormalization of tumor vessels [34]. Lov-induced downregulation of PlGF was validated at the mRNA (Table S2) and protein levels (Fig. 5C). VEGF mRNA levels were comparable in Vhcl- and Lov-treated tumors (Fig. 5D), suggesting that the Lov effect was specific for PlGF expression.

To further study the PlGF-producing cell types in the tumor parenchyma, we combined magnetic beads and FACS to isolate the three major cell populations in Tg-neu tumors, CD24^+^ tumor cells (luminal origin), CD31^+^ (endothelial cells) and CD45^+^ (hematopoietic cells). Lov treatment reduced PlGF mRNA levels in all three fractions (Fig. 5E), although this tendency was not statistically significant in any case. These results suggest that Lov downregulates PlGF by concerted inhibitory activity in the parenchymal and stromal compartments.

### Lovastatin shapes the inflammatory infiltrate towards an anti-tumor phenotype

**Figure 6:**
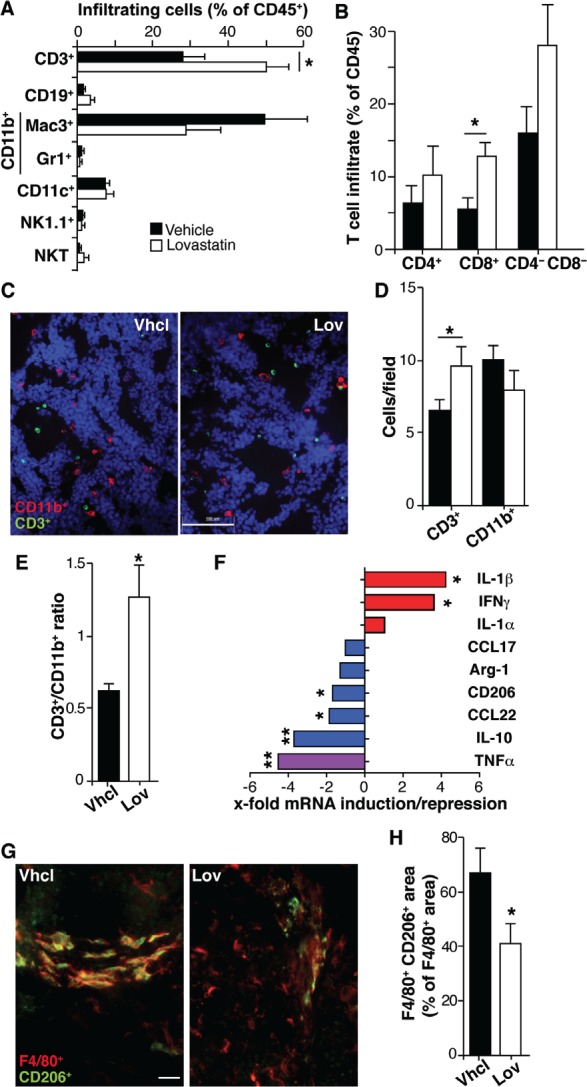
Lovastatin shapes the inflammatory infiltrate and reduces M2-like TAM polarization (A) Percentage of leukocyte subtypes that infiltrate Vhcl- and Lov-treated Tg-neu tumors. (B) Percentage of T lymphocyte subsets in tumors as in *A*. Mean ± SEM (*n* = 5 mice/condition). (C) Representative tumor sections stained for CD11b (myeloid cells, red) and CD3 (lymphoid cells, green); bar = 100 µm. (D) Quantification of myeloid and lymphoid cells from *C*. Mean ± SEM (*n* = 10 images from 2 tumors/group). (E) CD3^+^/CD11b^+^ cell ratio for *D*. (F) Lov-mediated induction or repression of mRNA for the indicated M1 (red) and M2 markers (blue) in isolated tumor-infiltrating CD45^+^ cells; the M1/M2 marker TNFα is indicated (violet). Values indicate the relative expression for Lov- and Vhcl-treated tumors, and represent the mean variation in mRNA expression from at least 4 tumors/group. (G) Representative sections from Tg-neu tumors in Vhcl- and Lov-treated mice, stained for the M2 marker CD206 (green) and the macrophage marker F4/80 (red); bar = 20 µm. (H) Quantification of M2-like TAM (F4/80^+^/CD206^+^) in images as in *G* (*n* = 20 images from 3 tumors/group). *B*, *D*, *E*, *F* and *H*, * p <0.05, **p<0.01, two-tailed Student's *t*-test.

Our results could explain the Lov effect on the tumor vasculature, but not the reduced tumor multiplicity associated with Lov treatment in Tg-neu mice (Fig. 1G). This prevention of tumorigenesis might be due to Lov-induced changes in inflammation and immune responses, two biological processes markedly affected by Lov treatment (Fig. 5B). We used FACS to analyze infiltration of Vhcl- and Lov-treated tumors by T (CD3^+^) and B cells (CD19^+^), TAM (Mac3^+^), granulocytes (Gr1^+^), dendritic (DC, CD11c^+^), natural killer (NK1.1^+^) and NK T cells (NK1.1^+^/CD3^+^). Tg-neu tumors in control mice were massively infiltrated by TAM (~50% of CD45^+^ cells) and to a lesser extent by T lymphocytes (~30%; Fig. 6A). Lov treatment significantly enhanced infiltration of T cells compared to TAM (Fig. 6A), particularly CD8^+^ T cells (Fig. 6B); Treg cell numbers were nonetheless unchanged in Vhcl- and Lov-treated tumors, as determined by FoxP3 mRNA analysis (not shown). Staining of tumor sections confirmed a significant increase in CD3^+^ cell infiltration in Lov-treated tumors (Fig. 6C, D), which reversed the T cell/TAM ratio in Vhcl-treated controls (Fig. 6E).

PlGF skews TAM towards a protumor, proangiogenic M2-like phenotype, which triggers angiogenesis and suppresses T cell-mediated immunity [30]. Since Lov downregulated PlGF in our model (Fig. 5), this treatment might also inhibit M2-like TAM polarization. M2-like TAM have high levels of mannose receptor-1 (MRC1/CD206), arginase-1 (Arg1), IL-10 and chemokines CCL22 and CCL17, whereas anti-tumor M1-like TAM express high interferon (IFN)γ and IL-1α/β levels; TNFα marks both M1- and M2-polarized TAM [6].

Analyses of purified tumor-infiltrating CD45^+^ cells indicated that Lov treatment increased mRNA levels for M1 markers, which was statistically significant for IFNγ and IL-1β, whereas it downregulated M2-like markers (with significant differences for IL-10, CD206 and CCL22; Fig. 6F). Lov treatment also reduced TNFα mRNA, which concurs with the reduced TAM infiltration in these tumors (Fig. 6F). CD206^+^ TAM number was lower in sections from Lov-treated tumors than those from controls (Fig. 6G, H), again indicating that Lov treatment prevented TAM polarization to the M2-like phenotype. Lov treatment thus increased effector T cell infiltration and prevented tumor-mediated skewing of TAM, two factors associated with enhanced anti-tumor immunity.

### Lovastatin inhibits growth of newly formed tumors only in immunocompetent hosts

**Figure 7:**
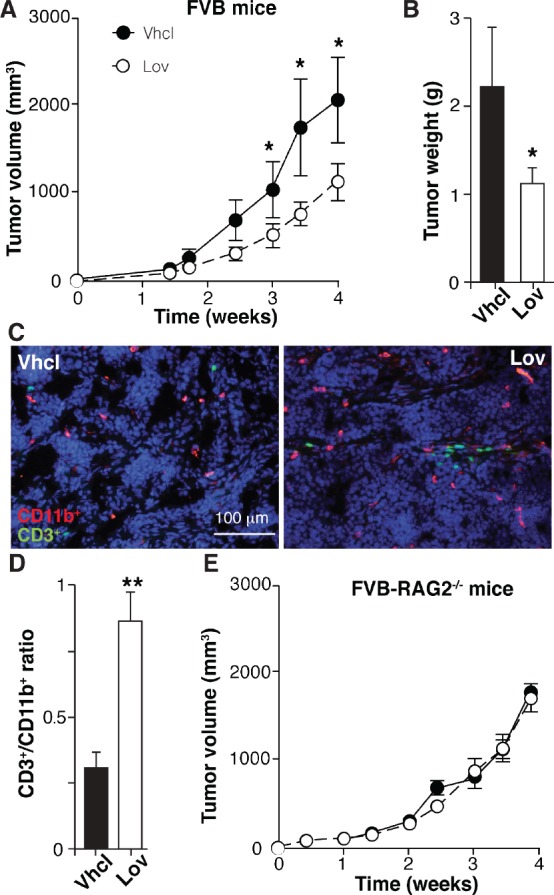
Lovastatin inhibits N202.1A tumor growth in immunocompetent but not in immunodeficient mice (A) Growth kinetics of N202.1A-induced tumors in immunocompetent Vhcl- or Lov-treated Tg-neu mice. (B) Weight of tumors in *A* after sacrifice. *A,B:* Values show mean ± SEM (*n* = 9 mice/group). (C) Representative sections from tumors in *A*, stained for CD11b (red) and CD3 (green). (D) CD3^+^/CD11b^+^ cell ratio in images as in *C* (*n* = 6 images/group). (E) Growth kinetics of N202.1A tumors implanted in immunodeficient Vhcl- or Lov-treated Rag2^−/−^ mice (*n* = 8 mice/group). *A*, *B*, *D*, * p <0.05, ** p<0.01, two-tailed Student's *t*-test.

Although Lov elicits a genetic program that fosters antitumor immunity, this treatment did not affect growth kinetics of established Tg-neu tumors (Fig. 1B). Tumor multiplicity was nonetheless reduced (Fig. 1G), suggesting that Lov treatment prevents growth of newly-formed tumors. To mimic this condition, we generated tumors by injecting the syngeneic N202.1A mammary tumor cell line into Tg-neu mice, and Lov treatment was initiated the same day. In this model, Lov administration significantly inhibited tumor growth kinetics as well as final tumor weight (Fig. 7A, B). As for spontaneous mammary tumors, Lov enhanced T cell infiltration into N202.1A-induced tumors (Fig. 7C), thus increasing the CD3^+^ cell/TAM ratio in Lov- compared to Vhcl-treated tumors (Fig. 7D). N202.1A tumor growth kinetics was independent of Lov treatment when cells were implanted in immunodeficient RAG2^−/−^ mice (Fig. 7E). This links the adaptive immune system to Lov-mediated inhibition of N202.1A tumor growth.

## DISCUSSION

A tumor-associated inflammatory response boosts incipient neoplasia acquisition of several cancer hallmarks, including angiogenesis and evasion of the immune system [35]. This inflammation is incongruous, since the immune cells responsible for surveillance to prevent nascent tumor development are co-opted to promote them. Reprogramming the tumor-induced inflammatory reaction to “immunological killing” activity could thus present a barrier to tumor formation and progression. Our results indicate that lovastatin is able to induce such reprogramming in spontaneous mammary tumors in Tg-neu mice.

Based on studies of numerous acute and chronic inflammation models, the Lov-induced changes in antitumor immunity in Tg-neu mice were unanticipated. A general phenomenon in these models is the Lov-induced shift from Th1 to Th2 responses (reviewed in [14]), which leads to upregulation of immunosuppressive cytokines (such as IL-10) and downregulation of those with antiviral and antitumor activities (such as IFNγ). In Tg-neu mice, however, Lov triggered IL-10 downregulation and IFNg upregulation in CD45^+^ cells, suggesting a bias toward a Th1 antitumor response. Statins are also reported to reduce Th2 cytokine levels in several models of Th2-mediated inflammation [36, 37]. The statins thus appear to promote a shift in the dominant immune phenotype, from Th1 to Th2 in autoimmune and acute inflammatory conditions, and from Th2 to Th1 in Th2- and tumor-associated inflammation.

Another striking observation in our study was the distinct Lov effect on tumor infiltration by lymphoid and myeloid cells. A consistent result of *in vivo* statin treatment is a decrease in leukocyte infiltration into inflamed tissue, through mevalonate-dependent and -independent mechanisms [14]. In late-stage Tg-neu tumors, Lov treatment did not alter total numbers of tumor-associated leukocytes (not shown), but selectively enhanced infiltration of cytotoxic CD8^+^ and to a lesser extent, of CD4^+^ and double-negative T cells. Lov also induced a consistent, although not significant, reduction of TAM infiltration into tumors; the mechanism that underlies this differential Lov effect on leukocyte infiltration requires further research. Some Lov-targeted molecules involved in transendothelial migration, including certain selectins and leukocyte integrins, participate in lymphoid and myeloid cell diapedesis [38]. Lov might also alter the expression of chemoattractants implicated in the specific infiltration of myeloid or lymphoid cells; for instance, Lov enhances specific infiltration of Treg cells into inflamed footpads by upregulating CCL1 expression in endothelial cells [31] as well as macrophage emigration from atherosclerotic plaques by upregulating CCR7 [39]. Expression profiling in whole tumors showed no Lov-regulated chemokines (Suppl. Table S1, S2). In the CD45^+^ cell fraction, Lov induced downregulation of CCL22, an attractant for Treg cells [40]; however, we found no differences in FoxP3 mRNA levels between Lov- and Vhcl-treated tumors.

Parallel to Lov-induced enhancement of T cell infiltration, we identified a role for this statin in TAM polarization. Analysis of tumor-infiltrating CD45^+^ leukocytes showed consistent Lov-mediated upregulation of IFNγ and IL-1β (M1 markers) and downmodulation of IL-10, CD206 and CCL22 (M2 markers). Moreover, microarray analysis showed Lov-induced downregulation of chitinase 3-like 3 (*Chi3l3, Ym1*), a gene associated to M2 macrophages [41]. This shift in macrophage polarization in Tg-neu tumors contrasts with statin potentiation of alternative M2 macrophage activation in experimental glomerulonephritis models [42] and in the vascular wall of patients with aortic aneurysm [43]. The Lov effect on the TAM phenotype is nonetheless atypical; Lov did not significantly alter IL-1α or IL-12 levels, which are associated with M1 polarization, or those of Arg-1 or CCL17, representative of the M2-like phenotype. It appears that Lov treatment does not cause full TAM reprogramming, but induces a specific genetic signature that reduces TAM differentiation to the pro-tumorigenic M2-like phenotype.

We propose that this M2-to-M1 shift in TAM polarization is mediated largely by Lov-induced downregulation of PlGF, a factor that drives TAM to the M2-like phenotype in tumors [30]. Since PlGF is also involved in aberrant tumor angiogenesis [34, 44], its downregulated expression in Lov-treated tumors might explain the improvement in perfusion and normalization of blood vessel structure. These changes in tumor vasculature indeed underlie the Lov-induced potentiation of the Doxo effect in Tg-neu tumors, as indicated by enhanced tumor parenchyma penetration by the cytotoxin. Based on these findings, we propose that improved vascular function and TAM bias away from the M2-like phenotype are mutual feedback processes, linked through PlGF downmodulation. Nevertheless, other Lov-induced changes in the tumor environment, including increased infiltration of immune effector cells, could contribute to tumor vessel normalization and the reprogramming of pro-tumorigenic TAM [45, 46].

An evident question is whether the Lov-induced changes in the inflammatory response are relevant to tumor biology. The combination of M1-biased TAM and the increase in T cell infiltration could be predicted to enhance antitumor immunity. The growth kinetics of established Tg-neu tumors was nonetheless unaltered by Lov administration, indicating that the effects are insufficient to overcome the immune editing induced by late-stage tumors. In contrast, Lov treatment effectively reduced the number of new spontaneous tumors in Tg-neu mice and inhibited N202.1A tumor growth in immunocompetent, but not in immunodeficient mice. We speculate that these alterations in immune system polarity have a protective function, which would explain the reduced tumor multiplicity in Lov-treated Tg-neu mice. A recent study associated statin use with improved progression-free survival in inflammatory breast cancer patients [47]. The role of statins in the prevention of human cancers is nevertheless debated [22, 48-50]. The partially protective effect of Lov on tumor onset in our murine model, suggests additional tumor mechanisms to bypass this preventive activity.

In summary, our findings indicate that statin treatment elicits a triple program that (1) improves vascular function, hence increasing the penetration of cytotoxic drugs into the tumor parenchyma, (2) enhances CD8^+^ T cell infiltration into the tumor, thus altering the effector:suppressor cell balance in the tumor stroma, and (3) re-educates TAM to an M1-like phenotype, which might rectify aberrant angiogenesis and create an environment prone to antitumor immunity rather than immune suppression. Our findings support the use of statins in cancer therapy, particularly in combination with immune-based strategies or drugs that induce immunogenic tumor cell death.

## MATERIALS AND METHODS

### Cell culture

N202.1A mammary cancer cells, derived from a Tg-neu tumor [51], were provided by Dr Vincenzo Bronte (Verona University, Italy) and cultured as described [29].

### Tumor induction and drug treatment

FVB/N-Tg(MMTVneu) 202Mul/J (Tg-neu) mice were from The Jackson Laboratory and Rag2^−/−^ FVB mice were described elsewhere [52]. Mammary tumors in Tg-neu mice were detected by weekly palpation, after which mice received a dose of Lov (10 mg/Kg, i.p.; Sigma-Aldrich) or Vhcl (5% ethanol) 3 times/week for 6 weeks (until killing). Where indicated, Lov treatment was combined with administration of doxorubicin (Doxo; 0.5 or 2.5 mg/Kg, 2 times/week, i.p.; Farmitalia Carlo Erba). N202.1A cells were inoculated s.c. in the right flank of the Tg-neu and Rag2^−/−^ mice (0.5 × 10^6^ cells); mice were treated with Lov or Vhcl from the day of cell injection, according to the schedule indicated above. Tumors were measured weekly (Tg-neu) or twice per week (N202.1A) with calipers and volume calculated (width^2^ × length/2). Live animal experiments were supervised by the CNB Ethics Committee, according to national and European Union guidelines.

### Apoptosis and proliferation analyses in mammary tumors

Cryopreserved tumor sections (10 µm) were acetone-fixed and stained with anti-phospho-histone H3 (#06-570; Millipore), followed by an amplification step with biotinylated secondary antibody and streptavidin-Cy3; 0.2% Triton X100 was included in all steps. For TUNEL, sequential tumor sections were fixed with 4% PFA (20°C) and stained with the MEBSTAIN Apoptosis kit II (MBL International).

### Hypoxia analyses

Necrotic areas were determined in sections (5 µm) from tumors fixed with neutral-buffered formalin (Sigma-Aldrich) before paraffin inclusion. Images of hematoxylin/eosin staining and autofluorescence, used for quantification (ImageJ, NIH), were acquired in a Leica (DM RB) microscope with a DP70 Olympus camera. Hypoxic areas were detected by injecting pimonidazole (Hypoxyprobe-1 Omni kit; Natural Pharmacia International) 30 min before killing mice by cervical dislocation, followed by staining with anti-pimonidazole antibody; hematoxylin was used to counterstain. Images were acquired as above, and quantified with Image-Pro Plus software. Tumor lactate levels were measured with the Lactate Colorimetric Assay kit (Abcam).

### Tumor perfusion and blood vessel parameters

Tumor-bearing mice were injected (i.v.) with FITC-lectin (100 µg; Vector Laboratories), killed after 10 min, and heart-perfused with 10% neutral-buffered formalin. Tumors were snap-frozen in tissue freezing medium (OCT; Jung) and 50 µm sections stained with anti-CD31 antibody (MEC13.3; BD Biosciences Pharmingen) and analyzed with a Radiance 2100 confocal system (BioRad) on an Axiovent 200 microscope (Zeiss). FITC-lectin^+^ and CD31^+^ vessels were determined with NIH-Image J software. Pericyte coverage was analyzed by staining with anti-CD31 and -nestin antibodies (ab6142; Abcam), and colocalization (Pearson's coefficient) determined with ImageJ (JACoP plug-in). Vessel number and area were quantified from anti-CD31-stained samples using ImageJ.

### Scanning electron microscopy (SEM)

Mice were perfused transcardially with heparinized saline (50 ml) and then with fixative (1% PFA, 1% glutaraldehyde in 0.1 M phosphate buffer; 100 ml) at a constant pressure of 120 mm Hg. Tumors were dissected out and immersed overnight in the same fixative (at 4% PFA, 4% glutaraldehyde). After several washes with PBS, each tumor was cut in half; one remained in the fixative mixture and the other was cryoprotected with 30% sucrose and frozen by immersion in dry ice-cooled isopentane. Frozen tumor pieces were fractured by striking a sharp blade placed on the specimen surface, then defrosted by immersion in chilled PBS. Frozen and non-frozen samples were postfixed in 1% osmium tetroxide in PBS (1 h), rinsed in distilled water, dehydrated in an acetone gradient, which was replaced with liquid carbon dioxide and completely evaporated by critical point drying. To enhance conductivity, samples were graphite-coated in a high vacuum evaporator and then gold-covered in a sputtering device. The vascular network was analyzed in a JEOL JSM 6400 scanning electron microscope.

### Doxorubicin quantification

Tumor extracts (100 mg) were homogenized in 0.5 ml PBS with daunorubicin (625 pmol) as internal standard. The homogenate (0.1 ml) was treated with 4 volumes of cold acetone and the mixture kept at −20°C for 150 min. After centrifugation of the precipitated proteins (16,000 *g*, 10 min), the organic solution was recovered and acetone evaporated under a nitrogen stream. The resulting residue was dissolved in the mobile phase (0.2 ml) and injected (0.1 ml) into an HPLC (Waters 2690 Alliance System) coupled to a Waters 2475 fluorescence detector (480 nm (excitation) and 560 nm (emission) wavelengths). Empower Software (Waters Corporation) was used for instrument control and data acquisition and processing. Molecules were separated on a C18 reverse-phase column (Kromasil 100 C18 15 x 0.4 cm, 0.5 µm; Teknokroma), with a 22:78 acetonitrile:water mixture, both containing 0.2% formic acid and 0.2% ammonium formate, as the mobile phase. Chromatographic areas corresponding to Doxo were compared to those of daunorubicin, and amounts were calculated from a calibration curve constructed by mixing different Doxo amounts (5, 10, 20, 40 and 80 pmol) with a fixed quantity of daunorubicin (40 pmol).

### Microarray analyses

Total RNA was obtained from Lov- and Vhcl-treated Tg-neu tumors (*n* = 5/treatment) using Tri-Reagent (Sigma-Aldrich) and further cleaned using RNeasy (Qiagen). RNA quality was confirmed by electropherogram analysis (Agilent 2100 Bioanalyzer). cDNA was synthesized from 4 µg total RNA using One-Cycle Target Labeling and Control Reagents (Affymetrix) to produce biotin-labeled cRNA. cRNA preparations (10 µg) were fragmented (94°C, 35 min) into 35- to 200-base fragments. Fragmented, biotin-labeled cRNA (10 µg) was hybridized to the Affymetrix Mouse Genome 430 2.0 GeneChip array containing 39,000 transcript variants from 34,000 well-characterized mouse genes. Each sample was added to a hybridization solution containing 100 mM 2-(N-morpholino)ethanesulfonic acid, 1 M Na^+^, and 20 mM EDTA with 0.01% Tween-20, to a final cRNA concentration of 0.05 µg/ml. After hybridization (16 h, 45°C), each microarray was washed and stained with streptavidin-phycoerythrin in a Fluidics station 450 (Affymetrix) and scanned at 1.56 µm resolution in a GeneChip Scanner 3000 7G System (Affymetrix).

Raw intensity values were summarized and normalized by the Robust Multi-array Analysis (RMA) algorithm [53]. After data processing, each probe was tested for expression changes over replicates using empirical Bayes moderated t-statistics [54]. To control the false discovery rate (FDR), p-values were corrected using the Benjamini-Hochberg method [55]. The FIESTA viewer (http://bioinfogp.cnb.csic.es/tools/FIESTA), developed by J.C. Oliveros (CNB Bioinformatics Core Facility), was used to visualize all microarray results and to evaluate the numerical thresholds applied for selecting differentially expressed genes. Original archives and normalized intensity values are deposited in the Gene Expression Omnibus database (NCBI-GEO; Acc. Code GSE42787, http://www.ncbi.nlm.nih.gov/geo/). Functional analyses of biological processes were determined by Genecodis, which indicates the significantly enriched gene ontology terms in the list of gene targets [56, 57].

### Quantitative PCR

Cell populations from tumors were purified as described [58]. Total RNA from total tumor samples or tumor cell fractions was extracted with Tri-Reagent or with the Easy RNA kit (Qiagen, when recovered cells ≤5 × 10^5^) and used to synthesize the first cDNA strand (High-capacity cDNA Archive Kit, Applied Biosystems) using random primers. Genes selected from the microarray analysis, and VEGF, cytokines, chemokines, and specific TAM markers were quantified by qRT-PCR in an ABI PRISM 7900HT System (Applied Biosystems) using a SYBR Green-based reaction mix (FluoCycle; EuroClone); β-actin amplification was used to normalize cDNA in each sample and to calculate ∆C_t_ values. Unless otherwise indicated, relative quantity (Rq) for each gene was calculated as 2^−∆∆Ct^ relative to the sample with the lowest expression.

### ELISA

PlGF levels were determined in RIPA (50 mM Tris-HCl, pH 8.0, with 150 mM NaCl, 1 mM EDTA, 1% NP40, 0.5% Na-deoxycholate, 0.1% SDS) tumor extracts with the mouse PlGF-2 immunoassay (R&D Systems).

### Determination of leukocyte infiltration and TAM polarization

Leukocyte infiltration was analyzed in Tg-neu tumors digested (90 min, 37°C) with collagenase P (1 mg/ml; Roche) and DNase I (100 µg/ml, Roche); cell suspensions were filtered through a 30 µm cell strainer and stained with anti-CD45 (clone I3/2.3), -CD19 (1D3), -Mac3, (M3/84), -NK1.1 (PK136) (BD Pharmingen), -CD3 (145-2C11), -CD8 (53-6.7), -CD11b (M1/70), -CD11c (N418) (eBioscience), -CD4 (GK1.5) and -Gr1 (RB6-8C5) (Beckman Coulter) and analyzed by FACS (Cytomics FC500, Beckman Coulter).

Immunohistochemistry was carried out in acetone-fixed, cryopreserved tumor sections (10 µm) stained with anti-CD3 (Dako), -CD11b (M1/70, Beckman Coulter), -F4/80 (BM8, eBioscience) and -CD206 (MR5D3, Serotec), followed by appropriate secondary antibodies or streptavidin-Cy3 (Jackson ImmunoResearch). Cells were counted and staining area determined with Image J. TAM polarization was quantified as the percentage of CD206^+^-stained area relative to that of F4/80^+^.

### Statistical analysis

Data represent mean ± SEM of replicate values from independent experiments. Statistical significance was calculated with the two-tailed Student's *t*-test or Mann-Whitney test for comparison between two groups. One-way ANOVA with Dunnett post-hoc tests was used for multiple comparisons. Differences were considered statistically significant when p <0.05.

## Supplementary Tables


