# Supplementary material for: A lovastatin-elicited genetic program inhibits M2 macrophage polarization and enhances T cell infiltration into spontaneous mouse mammary tumors
Source: Oncotarget. 2013 Oct 26;4(12):2288–301. doi: 10.18632/oncotarget.1376 (PMC3926827; doi:10.18632/oncotarget.1376)
Supplement: Supplementary file 1 [file oncotarget-04-2288-s001.doc]

*Suppl. Table S1****: Genes upregulated in Tg-neu tumors after lovastatin treatement***

| **Probe ID** | **Gene Symbol** | **Fold of variation (AFFYMETRIX)** | **p-VAL** | **Fold of variation (RT-qPCR)** |
| --- | --- | --- | --- | --- |
| 1452893_s_at | 1110065P19Rik | +3.37 | 0.00883930 |  |
| 1428739_at | 2310040A07Rik | +2.96 | 0.00960771 |  |
| 1453456_at | 2900084O13Rik | +2.04 | 0.01848227 |  |
| 1457671_at | 9330120H11Rik | +2.13 | 0.00487482 |  |
| 1455338_at | A4galt | +2.01 | 0.01611295 |  |
| 1434450_s_at | Adrbk2 | +2.55 | 0.00570424 | **n.d.** |
| 1451528_at | BC025833 | +2.28 | 0.01880700 |  |
| 1436789_at | Ccnjl | +2.63 | 0.00942679 |  |
| 1417649_at | Cdkn1c | +3.02 | 0.01218819 | +3.53 |
| 1444318_at | Chchd7 | +2.19 | 0.00310487 |  |
| 1425400_a_at | Cited4 | +2.16 | 0.01646017 | +2.23 |
| 1456877_at | Duox1 | +2.14 | 0.01192997 | **n.d.** |
| 1448507_at | Efhd1 | +5.29 | 0.01176520 |  |
| 1418569_at | Fblim1 | +2.22 | 0.00649830 |  |
| 1427443_at | Igkv14-111 | +2.17 | 0.00188028 |  |
| 1442368_at | Kctd12b | +2.42 | 0.01312430 |  |
| 1425078_x_at | LOC620078 | +2.94 | 0.01014131 |  |
| 1418061_at | Ltbp2 | +2.10 | 0.01061443 |  |
| 1419127_at | Npy | +12.58 | 0.00003405 | +12.56 |
| 1417526_at | Pcbp3 | +2.23 | 0.00478482 |  |
| 1452398_at | Plce1 | +2.32 | 0.00628965 |  |
| 1449876_at | Prkg1 | +2.45 | 0.01325143 |  |
| 1428268_at | Psd2 | +2.96 | 0.00005402 |  |
| 1455359_at | Ptpn14 | +2.22 | 0.01009810 |  |
| 1451362_at | Rab7l1 | +2.26 | 0.00177939 |  |
| 1424256_at | Rdh12 | +5.77 | 0.00676982 | +3.91 |
| 1440523_at | Rdhe2 | +2.25 | 0.01861623 |  |
| 1443832_s_at | Sdpr | +2.26 | 0.00851791 |  |
| 1455506_at | Slc25a34 | +2.25 | 0.01219438 |  |
| 1428642_at | Slc35d3 | +3.93 | 0.00158635 |  |
| 1418706_at | Slc38a3 | +2.14 | 0.01906393 |  |
| 1460129_at | Slc6a2 | +15.35 | 0.00083798 | +10.68 |
| 1417633_at | Sod3 | +3.08 | 0.01821658 | +2.90 |
| 1430567_at | Spink5 | +2.56 | 0.01232634 |  |
| 1423707_at | Tmem50b | +2.74 | 0.00022938 |  |
| 1419063_at | Ugt8a | +2.16 | 0.00361589 |  |
| 1420955_at | Vsnl1 | +2.65 | 0.00312430 |  |
| 1425098_at | Zfp106 | +2.03 | 0.00058966 |  |
| 1449314_at | Zfpm2 | +2.14 | 0.00506179 |  |

n.d., non-detected

***Suppl. Table S2: Genes downregulated in Tg-neu tumors after lovastatin treatement***

| **Probe ID** | **Gene Symbol** | **Fold of variation (AFFYMETRIX)** | **p-VAL** | **Fold of variation (RT-qPCR)** |
| --- | --- | --- | --- | --- |
| 1424518_at | 2310016F22Rik | -2.23 | 0.00519226 |  |
| 1435639_at | 2610528A11Rik | -3.83 | 0.01165076 |  |
| 1450387_s_at | Ak3l1 | -3.14 | 0.00849934 |  |
| 1418133_at | Bcl3 | -2.11 | 0.00256045 |  |
| 1422470_at | Bnip3 | -2.25 | 0.00113680 | **n.d.** |
| 1423954_at | C3 | -2.44 | 0.01987239 |  |
| 1436194_at | C330008K14Rik | -2.07 | 0.00780316 |  |
| 1421307_at | Car13 | -2.15 | 0.01587732 | -2.51 |
| 1417268_at | Cd14 | -2.60 | 0.01686902 |  |
| 1434376_at | Cd44 | -2.29 | 0.00908860 |  |
| 1423233_at | Cebpd | -2.70 | 0.00184921 |  |
| 1451537_at | Chi3l1 | -4.24 | 0.00354754 | -4.78 |
| 1460259_s_at | Clca1 | -11.98 | 0.00154993 | -18.53 |
| 1455232_at | Cml2 | -2.01 | 0.00836423 |  |
| 1448792_a_at | Cyp2f2 | -2.57 | 0.01026088 | -11.57 |
| 1459725_s_at | Dcpp1 | -24.80 | 0.00645913 | -30.33 |
| 1434580_at | Enpp4 | -2.54 | 0.00837424 |  |
| 1419029_at | Ero1l | -3.52 | 0.00262414 | -2.91 |
| 1416411_at | Gstm2 | -3.86 | 0.01805969 | -4.86 |
| 1422704_at | Gyk | -2.11 | 0.01194332 | -2.57 |
| 1418645_at | Hal | -2.04 | 0.00324816 |  |
| 1438037_at | Herc5 | -2.24 | 0.00302848 |  |
| 1424018_at | Hint1 | -2.15 | 0.00847301 |  |
| 1448239_at | Hmox1 | -2.64 | 0.01799435 | -2.89 |
| 1426278_at | Ifi27 | -3.54 | 0.00005370 |  |
| 1450783_at | Ifit1 | -2.44 | 0.00001674 | -2.81 |
| 1418293_at | Ifit2 | -2.38 | 0.00026362 |  |
| 1449025_at | Ifit3 | -2.84 | 0.00001199 | -3.4 |
| 1423754_at | Ifitm3 | -2.30 | 0.00070483 |  |
| 1447456_x_at | LOC671351 | -3.81 | 0.01337311 |  |
| 1426808_at | Lgals3 | -3.54 | 0.01109253 |  |
| 1417290_at | Lrg1 | -4.80 | 0.01397624 |  |
| 1438239_at | Mid1 | -2.05 | 0.01598880 |  |
| 1417256_at | Mmp13 | -2.25 | 0.00898137 | -3.62 |
| 1449587_a_at | Muc10 | -5.86 | 0.01043032 |  |
| 1451905_a_at | Mx1 | -2.51 | 0.00174636 | -4.0 |
| 1450977_s_at | Ndrg1 | -2.35 | 0.00060994 | -3.05 |
| 1420760_s_at | Ndrl | -3.58 | 0.00088923 |  |
| 1416432_at | Pfkfb3 | -2.08 | 0.01154500 | -2.32 |
| 1418471_at | Pgf | -2.75 | 0.00572188 | -2.86 |
| 1449184_at | Pglyrp1 | -4.14 | 0.00740642 |  |
| 1437893_at | Plb1 | -3.10 | 0.00735658 |  |
| 1420388_at | Prss12 | -2.01 | 0.01183608 |  |
| 1436058_at | Rsad2 | -3.14 | 0.00005334 | -3.32 |
| 1419100_at | Serpina3n | -2.01 | 0.00399102 |  |
| 1415802_at | Slc16a1 | -2.82 | 0.00295649 |  |
| 1416342_at | Tnc | -2.85 | 0.01224459 | -3.73 |
| 1418345_at | Tnfsf13 | -2.68 | 0.01093668 | -2.81 |
| 1418191_at | Usp18 | -3.04 | 0.00076537 |  |

n.d., non-detected
